# The East London Hospital for Children

**Published:** 1893-12-02

**Authors:** 


### THE EAST LONDON HOSPITAL FOR CHILDREN.

At the meeting of this institution held last week, Mr. H. W. Trinder, Chairman of the Board of Management, presided, and the Earl of Erroll, Major J. Roper Parkington, Rev. Father Gorman and other governors were present. In moving the adoption of the report, the Chairman mentioned, and hoped it would be made known, that they had only £200 at their credit, with which they had to finish the year. The report of the Institution showed that this hospital has not been exempt from the pressure of hard times, which all charities have been feeling. The report, which relates to the period of six months that ended on June 30th, shows that while the work of the hospital has increased by about 25 per cent., the income has decreased by over 25 per cent. The receipts during this period were £4,692 as compared with £7,632 for the corresponding period of 1892, while the new patients, "in" and "out," were for the first six months of 1893, 13,777, as against 9,557 in the corresponding months of 1892. So far, however, it is gratifying to state that no debt has been incurred on account of the general maintenance of the hospital. In connection with the building of the new out-patients' room a debt of £2,000 still remains, which the board are very anxious should be cleared off as soon as possible. Negotiations are in progress with a view to securing a site for a convalescent home on the East Coast. Towards the building of this home a fund of £3,310 has been raised. The board make special mention of the generosity of Mr. Charles A. Prescott, who in August last supplemented many former munificent gifts to the hospital by presenting it with £5,000. Mr. R. O. Howlett had a grievance to ventilate, and in doing so read extracts from a lengthy letter he had received from a woman who had gone to the hospital as an out-patient, and had been kept waiting for two or three hours. The Chairman and others pointed out to Mr. Howlett that the delay was unavoidable. In order to cope with the large and increasing out-patient department, it had been decided to increase the number of the visiting medical staff. Mr. Charles Cheston has been elected one of the vice-presidents; and the names of Mr. Cheston and Mr. Trinder were added to the list of trustees.

